# Evaluation of Serum Biomarkers for Patients at Increased Risk of Stroke

**DOI:** 10.1155/2012/906954

**Published:** 2012-03-15

**Authors:** Pelisek Jaroslav, Reeps Christian, Ockert Stefan, Zimmermann Alexander, Peter Zepper, Poppert Holger, Eckstein Hans-Henning

**Affiliations:** ^1^Clinic of Vascular Surgery, Klinikum Rechts der Isar der Technischen Universitaet Muenchen, Ismaninger Straße 22, 81675 Munich, Germany; ^2^Department of Neurology, Klinikum Rechts der Isar der Technischen Universitaet Muenchen, Germany

## Abstract

Early recognition of vulnerable patients is an important issue for stroke prevention. In our study, a multiscore analysis of various biomarkers was performed to evaluate its superiority over the analysis of single factors. Study subjects (*n* = 110) were divided into four groups: asymptomatic patients with stable (*n* = 25) and unstable (*n* = 36) plaques and symptomatic patients with stable (*n* = 13) and unstable (*n* = 36) plaques. Serum levels of MMP-1, -2, -3, -7, -8, -9, TIMP-1, -2, TNF-*α*, IL-1b, and IL-6, -8, -10, -12 were measured. Multi-score analysis was performed using multiple receiver operating characteristics (ROC) and determination of appropriate cutoff values. Significant differences between the groups were observed for MMP-1, -7, -9 and TIMP-1 in serum of the study subjects (*P* < 0.05). Multiple biomarker analysis led to a significant increase in the AUC (area under curve). In case of plaque instability, positive predictive value (PPV) for up to 86.4% could be correctly associated with vulnerable plaques. Thus, multiscore analysis might be preferable than the use of single biomarkers.

## 1. Introduction

Early recognition of rupture-prone atherosclerotic lesions in patients with high-graded carotid artery stenosis is an important clinical issue to prevent ischemic stroke [1–5]. Various pathophysiological mechanisms are responsible for the plaque progression and vulnerability such as degradation of extracellular matrix components especially by matrix metalloproteinases (MMPs), intensified inflammatory reaction, and neovascularisation [3, 5–7]. These features are the main reason for plaque rupture and consequent neurological symptoms. Thus, MMPs and inflammatory factors might also serve as possible markers for patients with unstable high-graded carotid artery stenosis [2, 8–16]. However, the data that have been achieved up to date are not consistent. Some studies investigated patients with symptomatic versus asymptomatic carotid stenosis or patients with or without emboli [12, 14–17]. Other researchers compared stable versus unstable plaques [2, 18, 19]. Furthermore, only very few investigations evaluated the usefulness of multiple biomarkers to predict rupture-prone atherosclerotic lesions [2, 17, 20, 21].

The aim of this work was the comparison of results of multiple analyses of various relevant biomarkers in patients with stable versus unstable carotid plaques and in individuals with or without neurological symptoms to evaluate whether multiple-score evaluation is superior to the analysis of single factors.

## 2. Materials and Methods

### 2.1. Study Patients

The retrospective study consisted of 110 consecutive patients with high-grade carotid artery stenosis >70% (determined by ultrasound) [22], intended for carotid endarterectomy (CEA). All patients underwent a detailed neurological examination by a neurologist, and the carotid plaques were analysed by means of histology to divide the study subjects into four groups: (1) asymptomatic patients with stable plaques (*n* = 25); (2) asymptomatic patients with unstable plaques (*n* = 36); (3) symptomatic patients with stable plaques (*n* = 13); (4) symptomatic patients with unstable plaques (*n* = 36). The study was performed according to the Guidelines of the World Medical Association Declaration of Helsinki.

### 2.2. Histological Characterisation of Carotid Artery Lesions

The excised carotid plaques were fixed in formalin, separated into segments of 3-4 mm, and embedded in paraffin. From each segment sections of 2-3 *μ*m were prepared and routinely stained with hematoxylin/eosin (HE) and Elastin van Gieson (EvG) to assess the morphological and histological features of each plaque. Stained specimens were analysed by light microscopy from two independent and experienced investigators blinded for the study groups. In accordance with our own expertise and the study of Rothwell and Redgrave [18, 19], appropriate selection into stable or unstable plaques was performed.

### 2.3. Immunohistochemistry (IHC)

Immunohistochemical analyses were performed on formalin-fixed, paraffin-embedded carotid plaques. Primary antibodies against following antigens were used: CD68 (macrophages/monocytes; Dako), CD45 (inflammatory infiltrates; Novocastra, UK), smooth muscle cell actin (Dako, Hamburg, Germany), and Factor VIII (endothelial cells; Dako). Visualisation was performed with Peroxidase/DAB ChemMate Detection Kit (Dako).

### 2.4. Blood Sample Analyses

Blood sampling was performed from all patients immediately prior to surgical intervention. MMPs and TIMPs were quantified in serum samples using ELISA assays from R&D System (Quantikine human MMPs and TIMPs kit; Wiesbaden-Nordenstadt, Germany) according to the manufacture's protocols. The colour development was determined by multiplate reader Mithras LB940 (Berthold Technologies, Bad Herrenalb, Germany) at 450 nm with correction at 570 nm. Inflammatory markers were analysed using Cytometric Bead Array (BD Biosciences, San Jose, CA, USA). Fibrinogen activity was determined by the Clauss method (Dade Behring, Schwalbach, Germany). The hsCRP (high sensitivity CRP) was determined by ELISA assay (Life Diagnostics; West Chester, PA, USA. Additional clinical blood parameters were measured in laboratories of our clinical chemistry.

### 2.5. Statistical Analyses

Statistical analyses were performed with SPSS for Windows version 17.0. (SPSS Inc., Chicago, IL, USA). Values of continuous variables were expressed as mean ± standard deviation (SD). Kolmogorov-Smirnov test was used to assess normal distribution. One-way ANOVA test was applied for comparison between the groups. Correlations between the single factors were quantified by Spearman's rank correlation coefficient. Receiver operating characteristics (ROC) analysis was applied to evaluate the optimal positive and negative predictive value of each prognostic marker and their combinations. DeLong and Clarke-Pearson approach was used to compare ROC curves to provide the best statistical evidence [23]. All statistical comparisons were performed two sided in sense of an exploratory data analysis using *P* < 0.05, *P* < 0.01, and *P* < 0.001 as level of significance.

## 3. Results

### 3.1. Patient Characteristics

The demographic data of all patients are summarised in Table 1. All groups were well matched, without any significant differences with regard to patient epidemiology, associated diseases, or medication. The average age of the study population was 69 years (range 59 to 79). The majority of patients had hypertension (>87%) and about one-third suffered under accompanying coronary heart disease. All patients with the exception of one individual received ASA or clopidogrel, and more than half of the study subjects were on statins.

### 3.2. Serum Levels of MMPs, TIMPs, and Inflammatory Factors

The results of blood serum analysis are summarised in Table 2. Significant differences between the groups were observed only for MMP-1, -7, -9, and TIMP-1. (*P* = 0.047, 0.005, 0.028, and 0.044, resp.). Tendency was observed also for MMP-8; the difference was, however, not statistically significant. In many cases, increased level of various inflammatory factors was found in the group of symptomatic patients with unstable carotid plaques. However, again the values were not statistically different.

### 3.3. Correlation Analysis

Regarding causal relationships between the individual factors analysed in our study, we performed correlation analysis between MMPs, TIMPs, and all the inflammatory factors tested in blood of the patient cohort (Table 3). Most correlations were found between individual MMPs and TIMPs: the levels of MMP-1 correlated significantly with MMP-7 and TIMP-1 (*P* < 0.001 and *P* < 0.05, resp.), MMP-2 with TIMP-2 (*P* < 0.001), MM-3 with MMP-7 (*P* < 0.05), MMP-7 with TIMP-1 (*P* < 0.001), MMP-8 with MMP-9 and TIMP-1 (*P* < 0.001 and *P* < 0.05, resp.), and TIMP-1 with TIMP-2 (*P* < 0.001). Furthermore, significant correlations were observed between some inflammatory factors and MMPs: IL-1*β* correlated with MMP-9 (*P* < 0.05), IL-6 with MMP-7 and -8 (*P* < 0.001 and *P* < 0.01, resp.), and IL-12 with MMP-2 (*P* < 0.01); the amount of leukocytes was related to MMP-8 and -9 (*P* < 0.01 and *P* < 0.001, resp.) and fibrinogen to MMP-7, -8, and -9 (*P* < 0.05 and *P* < 0.001, resp.).

### 3.4. Multiple ROC Curve Analysis

To evaluate whether the individual factors and their appropriate combination in blood of the study patients can be associated with carotid plaque instability or neurological symptoms, ROC curve was designed accordingly. Furthermore, multiple analysis was performed to assess the validity of biomarkers to further improve the predictability. In accordance with the above-described analyses between the study groups, only factors with significant differences were involved in the ROC curve analysis: MMP-1, -7, -9, and TIMP-1. The area under curve (AUC) and the predictive values are summarised in Table 4. The cutoffs were selected by using DeLong and Clarke-Pearson approach to provide the best statistical evidence [23]. All patients with biomarker values higher than the ascertained cutoff points were considered as individuals with neurological symptoms or as patients with unstable plaques. Furthermore, multiple ROC analysis was performed for all possible combinations of the above-described biomarkers. Regarding neurological symptoms, the positive predictive value (PPV) was quite low with around 50% for the single factors and increased over 60% by their appropriate combination. In contrast, the negative predictive value (NPV) was significantly higher with curtly under 70% for the individual biomarkers and achieving up to 80% for the factor grouping. With regard to the maximal PPV, combination of MMP-7 and TIMP-1 achieved the highest value of 65.1%. On the contrary to the neurological symptoms, PPV for patients with vulnerable plaques was markedly higher with 76–79% for the single factor analysis. Their grouping led to an increase for up to 86.4% using combined analysis with MMP-1, -7, and -9. The NPV remained almost unchanged independent of the combination used. 

## 4. Discussion

Biomarkers for prevention of stroke are promising diagnostic tool in medical praxis. Due to the heterogeneity of the atherosclerotic lesions, however, single marker will never be sufficient for reliable prediction of patients at increased risk of stroke. Furthermore, the values of the biomarkers and the cutoff value at risk can markedly alternate between the individual patients. So far only few studies have evaluated the usefulness of multiple biomarkers and their overall association with neurological symptoms or plaque vulnerability, especially concerning unstable carotid lesions. 

From the various MMPs, their inhibitors, and the inflammatory factors analysed in this study, serum levels of MMP-1, -7, -9, and TIMP-1 were significantly increased in patients with neurological symptoms and vulnerable plaques. All these factors were already described being involved in the degradation of extracellular matrix [5, 7, 10, 11, 24, 25]. Thus, they could reliably reflect unstable carotid plaques in patients at risk of stroke. Interestingly, different biomarkers can be related either to neurological symptoms or to plaque instability [2, 17]. This is an important issue, because many researchers often equate neurological symptoms with plaque vulnerability. However, not all ischemic strokes are caused by unstable plaques. Only 15–20% of all ischemic strokes account for atherosclerotic carotid stenosis [5, 26]. Therefore, prevention of stroke on the basis of biomarker evaluation in the blood of the concerned patients should be related to plaque vulnerability. This is the reason for the discrepancy between the individual biomarker in our study related either to neurological symptoms or carotid plaque stability. From our point of view, the markers that show significant differences between patients with stable versus unstable plaques are of higher value compared to biomarkers corresponding with symptoms. This was also the reason why we have combined neurological symptoms and plaque vulnerability together. Interestingly, various MMPs and inflammatory factors could be associated with different clinical findings. MMP-1, -7, and TIMP-1 were associated with either symptoms or plaque instability. In contrast, MMP-9 seemed to be related mainly to the neurological symptoms (Table 2). Regarding inflammatory factors, increased levels of TNF-*α* and IL-6 were associated with plaque instability, IL-10 only in the group of symptomatic patients with unstable plaques. High sensitive CRP appeared to be more related to neurological symptoms than to plaque instability and fibrinogen for both. Again, these data confirm the pathophysiological differences between patient symptomatic and plaque vulnerability [2, 17]. Nevertheless, our data demonstrated that whether the symptoms are caused by unstable carotid plaques or atherosclerotic changes in other vessels can also have common features that can be reflected in the blood of patients at risk of stroke. This assumption was further confirmed by the correlation analysis of the individual factors (Table 3). 

Interestingly, independent of neurological symptom or plaque instability, no statistically significant differences between patients with stable and unstable lesions were observed for MMP-2 as described in some former studies [13–15]. The reason for these discrepancies is that we have characterised the plaques in accordance with Redgrave and Rothwell [18, 19], looking effectively for the true unstable plaques. In contrast, the early studies graded the plaques according to AHA classification, assuming atherosclerotic lesion type V as stable, type VI as unstable. But patients with carotid lesions of type VI (plaques with thrombotic deposits and haemorrhage) with thick fibrous cap can still be considered as stable. And plaques of type V with thin fibrous cup under 200 *μ*m over a large necrotic core can be considered as unstable. 

Regarding inflammatory factors, no significant differences were observed between the groups, even if some tendencies have been observed, especially for TNF-*α*, IL-6, IL-10, CRP, and fibrinogen. However, it is to consider that our patients had already an advanced stage of carotid artery stenosis >70% and almost all of them were hypertensive. So, the level of many of the inflammatory factors in blood of our patients was already increased, compared to healthy individuals. In addition, atherosclerotic lesions are frequently accompanied by chronic inflammation. So, inflammatory factors may correlate with advanced carotid stenosis, are, however, not specific enough to detect vulnerable plaques [25, 27]. 

The main goal of the study was to evaluate whether combination of relevant biomarkers of advanced carotid lesions might be better associated with neurological symptoms or plaque vulnerability compared with single biomarkers. The combination of selected biomarkers led indeed to increased AUC and PPVs. Interestingly, high positive prediction could be associated with plaque vulnerability for up to 86% compared to only 65% regarding patient symptomatic. In contrast, negative prediction was high for the clinical symptoms with up to 80%, but the factor grouping did not significantly improve the NPV for patients with unstable plaques, which was in the most cases between 50 and 60%, considering patients incorrectly as individuals at risk. The problem of biomarkers is generally that they often influence each other. So, if the level of one of them is increased, the others are increased as well (see also Table 3). The combination of such biomarkers, however, does not necessarily lead to an increased predictivity over the analysis of single factors. To establish a set of relevant biomarkers for more reliable diagnosis of stroke, independent markers are necessary to improve the overall sensitivity and especially specificity [20]. Still, our data demonstrate that the use of more than one biomarker better correlates with the clinical findings and seems therefore to be superior to the analysis of single factors. These results further confirm our assumption that multivariate analyses of relevant biomarkers are necessary to reduce the risk of inaccurate diagnosis. 

## 5. Conclusion

In summary, we evaluated various predictive biomarkers and their combinations in patients with advanced carotid stenosis in order to improve the concordance with clinical findings such as neurological symptoms or/and unstable plaques. Further experiments, especially large prospective clinical studies, are necessary to evaluate the relevance of such biomarkers. In addition, the proper cutoff values have to be accurately interpreted and carefully reevaluated before they can be used in clinical praxis for, for example, reliable prevention of patients at increased risk of stroke. 

## Figures and Tables

**Table 1:** Patients characteristics.

	Asym/stable*	Asym/unstable	Sym/stable*	Sym/unstable	*P* value**
	*n* = 25 of 110	*n* = 36 of 110	*n* = 13 of 110	*n* = 36 of 110	
Age (years)	68.8 ± 9.7	67.6 ± 7.7	71.6 ± 7.8	68.6 ± 9.6	0.210
Sex (male/female)	14/11	29/7	9/4	22/24	0.140

Associated diseases					

Coronary heart disease	40.0	46.8	27.3	30.3	0.491
Hypertension	90.5	87.5	90.9	78.8	0.586
Chronic kidney disease	4.8	9.3	9.1	3.1	0.724
Diabetes mellitus	14.3	37.5	27.3	18.2	0.106

Medication					

ASA/clopidogrel	100.0	96.8	100.0	100.0	0.573
Beta-blockers	65.0	74.2	72.7	65.6	0.860
ACE inhibitors	35.0	51.6	45.4	46.9	0.322
Statins	70.0	54.8	54.5	64.1	0.212

Age: mean ± standard deviation; all other values are in % of the study subjects within the each group; ASA: Acetyl salicylic acid.

*Asym: asymptomatic patients, Sym: symptomatic patients.

**One-way ANOVA (analysis of variance) was used.

**Table 2:** Levels of various clinical factors in blood serum of study subjects.

	Asym/stable	Asym/unstable	Sym/stable	Sym/unstable	*P* value**
	*n* = 25 of 110	*n* = 36 of 110	*n* = 13 of 110	*n* = 36 of 110	
MMP-1 [ng/*μ*L]	2.4 ± 1.8	4.4 ± 3.8	4.1 ± 2.8	3.9 ± 2.7	**0.047**
MMP-2 [ng/*μ*L]	257 ± 81	261 ± 91	237 ± 62	269 ± 81	0.672
MMP-3 [ng/*μ*L]	14.1 ± 5.5	14.8 ± 8.7	14.6 ± 4.1	13.4 ± 4.7	0.279
MMP-7 [ng/*μ*L]	7.1 ± 3.2	10.1 ± 4.7	11.6 ± 5.2	10.7 ± 4.1	**0.005**
MMP-8 [ng/*μ*L]	11.3 ± 9.1	11.2 ± 11.5	10.2 ± 6.5	17.1 ± 19.5	0.212
MMP-9 [ng/*μ*L]	192 ± 65	178 ± 74	210 ± 66	231 ± 80	**0.028**
TIMP-1 [ng/*μ*L]	139 ± 41	161 ± 70	167 ± 47	182 ± 59	**0.044**
TIMP-2 [ng/*μ*L]	65.0 ± 22.2	69.0 ± 29.1	61.1 ± 29.6	74.5 ± 41.7	0.552

TNF-*α* [pg/*μ*L]	2.5 ± 0.8	3.2 ± 1.2	2.3 ± 1.4	3.3 ± 1.5	0.134
IL-1*β* [pg/*μ*L]	1.1 ± 1.4	2.1 ± 2.7	1.8 ± 1.6	2.5 ± 4.1	0.523
IL-6 [pg/*μ*L]	2.2 ± 1.2	3.3 ± 1.9	2.8 ± 1.1	3.9 ± 2.8	0.397
IL-8 [pg/*μ*L]	6.5 ± 1.4	6.1 ± 2.7	5.2 ± 2.7	6.8 ± 5.3	0.482
IL-10 [pg/*μ*L]	1.4 ± 0.9	1.8 ± 0.7	1.6 ± 1.1	3.0 ± 2.7	0.234
IL-12 [pg/*μ*L]	1.1 ± 0.6	1.9 ± 1.7	2.1 ± 1.4	2.8 ± 2.8	0.683

hsCRP [mg/dL]	1.1 ± 1.2	1.2 ± 0.7	2.3 ± 2.8	2.1 ± 1.6	0.060
Fibrinogen [mg/dL]	345 ± 94	371 ± 122	382 ± 65	361 ± 83	0.790
Leukocytes [mg/dL]	7.1 ± 1.4	7.5 ± 1.9	8.1 ± 1.4	7.1 ± 2.0	0.334

**One-way ANOVA (analysis of variance) was used.

**Table 3:** Correlation analysis for various blood parameters.

	MMP-1	MMP-2	MMP-3	MMP-7	MMP-8	MMP-9	TIMP-1	TIMP-2
MMP-1	—							
MMP-2	n.c.	—						
MMP-3	n.c.	n.c.	—					
MMP-7	0.324***	n.c.	0.264*	—				
MMP-8	n.c.	n.c.	n.c.	n.c.	—			
MMP-9	n.c.	n.c.	n.c.	n.c.	0.746***	—		
TIMP-1	0.195*	n.c.	n.c.	0.315***	0.225*	n.c.	—	
TIMP-2	n.c.	0.531***	n.c.	n.c.	n.c.	n.c.	0.370***	—

IL1-*β*	n.c.	n.c.	n.c.	n.c.	n.c.	0.227*	n.c.	n.c.
IL-8	n.c.	n.c.	n.c.	0.328***	0.298**	n.c.	n.c.	n.c.
IL-12	n.c.	−0.287**	n.c.	n.c.	n.c.	n.c.	n.c.	n. c.

Leukocytes	n.c.	n.c.	n.c.	n.c.	0.275**	0.366***	n.c.	n.c.
CRP	n.c.	n.c.	n.c.	n.c.	n.c.	n.c.	n.c.	n.c.
Fibrinogen	n.c.	n.c.	n.c.	0.274*	0.428***	0.418***	n.c.	n.c.

*<0.05, **<0.01, ***<0.001.

**Table 4:** Selected values of positive and negative predictive value (PPV, NPV) leading to improved prognosis of patients with either neurological symptoms or plaque instability.

	Neurol. symptoms			Plaque instability		
	AUC*	PPV*	NPV*	AUC*	PPV*	NPV*
MMP-1	n.c.	n.c.	n.c.	0.691	79.6	55.3
MMP-7	0.639	49.3	69.2	0.615	76.2	45.8
MMP-9	0.637	52.5	67.5	0.674	79.2	50.9
TIMP-1	0.651	56.4	64.9	0.654	79.1	48.3

MMP-1 + MMP-7	n.c.	n.c.	n.c.	n.c.	n.c.	n.c.
MMP-1 + MMP-9	n.c.	n.c.	n.c.	n.c.	n.c.	n.c.
MMP-1 + TIMP-1	n.c.	n.c.	n.c.	n.c.	n.c.	n.c.
MMP-7 + MMP-9	0.684	54.9	80.1	0.704	81.6	53.8
MMP-7 +TIMP-1	0.681	65.1	68.9	0.698	81.6	53.8
MMP-9 + TIMP-1	0.687	62.2	69.6	0.713	83.7	55.8

MMP-1 + MMP-7 + MMP-9	0.661	62.2	69.6	0.714	86.4	54.4
MMP-1 + MMP-7 + TIMP-1	0.655	61.4	68.4	0.727	79.7	59.5
MMP-1 + MMP-9 + TIMP-1	0.667	60.8	72.1	n.c.	n.c.	n.c.
MMP-7 + MMP-9 + TIMP-1	0.707	61.1	74.5	0.723	80.1	52.9

MMP-1 + MMP-7 + MMP-9 + TIMP-1	0.672	60.2	73.9	0.729	86.4	54.4

*AUC: area under curve; only values with statistical significance are shown; n.c.: no further increase in positive (PPV) and negative (NPV) predictive value.
